# Supplementary material for: Content-rich biological network constructed by mining PubMed abstracts
Source: BMC Bioinformatics. 2004 Oct 8;5:147. doi: 10.1186/1471-2105-5-147 (PMC528731; doi:10.1186/1471-2105-5-147)
Supplement: Additional File 2 — The original results of the above study (non-essential files are deleted to keep the file size under the limit set by BMC bioinformatics). [file 1471-2105-5-147-S2.bz2 › chilibotAdditionalFile2/dip05/664ID9346309E11057/html/CCR3_CCL15.html]

 


 **CCR3** and **CCL15** 
  
Found 8 abstracts in PubMed, retrieved 05.  
 

 What does Google say? 
 PDF only 
| .edu only 

---

**Interactive relationship** (e.g. stimulation, inhibition, etc)

**Neutral relationship**- Leukotactin 1  [ **CCL15** ]  Lkn 1  [ **CCL15** ]   **CCL15**  is a recently cloned CC chemokine that binds to the CCR1 and  **CCR3** .  Ref: 11943214 FEBS Lett, 2002
- Leukotactin 1  [ **CCL15** ]  Lkn 1  [ **CCL15** ]  is a human CC chemokine that binds to both CC chemokine receptor 1 CCR1 and  **CCR3** .  Ref: 11832479 J Biol Chem, 2002
- In contrast,  **CCL15**  is characterized using molecular forms deduced from the mRNA cDNA and shown to activate cells via CCR1 and  **CCR3** , also dependent on the amino terminal length.  Ref: 11527984 J Leukoc Biol, 2001
- The human CC chemokine leukotactin 1  [ **CCL15** ]  Lkn 1  [ **CCL15** ]  is both a strong chemoattractant for neutrophils, monocytes, and lymphocytes and a potent agonist for CCR1 and  **CCR3** .  Ref: 10202040 J Immunol, 1999
- Human CC chemokine 2 HCC 2  [ **CCL15** ]  is a novel member of the chemokine peptide family that induces chemotaxis of monocytes, T lymphocytes and eosinophils via activation of the CCR 1 and CCR 3  [ **CCR3** ]  receptors.  Ref: 10604595 J Pept Res, 1999

**Non-interactive relationship** (e.g. studied together, co-existance, homology, etc.)

- In  **CCR3**  expressing cells, Delta24 was the only form of Lkn 1  [ **CCL15** ]  mutants that revealed increased agonistic potency.  Ref: 11832479 J Biol Chem, 2002
